# Adverse drug reactions caused by drug-drug interactions reported to Croatian Agency for Medicinal Products and Medical Devices: a retrospective observational study

**DOI:** 10.3325/cmj.2011.52.604

**Published:** 2011-10

**Authors:** Nikica Mirošević Skvrce, Viola Macolić Šarinić, Iva Mucalo, Darko Krnić, Nada Božina, Siniša Tomić

**Affiliations:** 1 Pharmacovigilance Department, Agency for Medicinal Products and Medical Devices, Zagreb, Croatia; 2 Centre for Applied Pharmacy, Faculty of Pharmacy and Biochemistry, University of Zagreb, Zagreb, Croatia; 3 Department of Laboratory Diagnostics, University School of Medicine and University Hospital Center, Zagreb, Croatia; 4 Agency for Medicinal Products and Medical Devices, Zagreb, Croatia

## Abstract

**Aim:**

To analyze potential and actual drug-drug interactions reported to the Spontaneous Reporting Database of the Croatian Agency for Medicinal Products and Medical Devices (HALMED) and determine their incidence.

**Methods:**

In this retrospective observational study performed from March 2005 to December 2008, we detected potential and actual drug-drug interactions using interaction programs and analyzed them.

**Results:**

HALMED received 1209 reports involving at least two drugs. There were 468 (38.7%) reports on potential drug-drug interactions, 94 of which (7.8% of total reports) were actual drug-drug interactions. Among actual drug-drug interaction reports, the proportion of serious adverse drug reactions (53 out of 94) and the number of drugs (n = 4) was significantly higher (*P* < 0.001) than among the remaining reports (580 out of 1982; n = 2, respectively). Actual drug-drug interactions most frequently involved nervous system agents (34.0%), and interactions caused by antiplatelet, anticoagulant, and non-steroidal anti-inflammatory drugs were in most cases serious. In only 12 out of 94 reports, actual drug-drug interactions were recognized by the reporter.

**Conclusion:**

The study confirmed that the Spontaneous Reporting Database was a valuable resource for detecting actual drug-drug interactions. Also, it identified drugs leading to serious adverse drug reactions and deaths, thus indicating the areas which should be in the focus of health care education.

Adverse drug reactions (ADR) are among the leading causes of mortality and morbidity responsible for causing additional complications (1,2) and longer hospital stays. Magnitude of ADRs and the burden they place on health care system are considerable (3-6) yet preventable public health problems (7) if we take into consideration that an important cause of ADRs are drug-drug interactions (8,9). Although there is a substantial body of literature on ADRs caused by drug-drug interactions, it is difficult to accurately estimate their incidence, mainly because of different study designs, populations, frequency measures, and classification systems (10-15).

Many studies including different groups of patients found the percentage of potential drug-drug interactions resulting in ADRs to be from 0%-60% (10,11,16-25). System analysis of ADRs showed that drug-drug interactions represented 3%-5% of all in-hospital medication errors (3). The most endangered groups were elderly and polimedicated patients (22,26-28), and emergency department visits were a frequent result (29). Although the overall incidence of ADRs caused by drug-drug interactions is modest (11-13,15,29,30), they are severe and in most cases lead to hospitalization (31,32).

Potential drug-drug interactions are defined on the basis of on retrospective chart reviews and actual drug-drug interactions are defined on the basis of clinical evidence, ie, they are confirmed by laboratory tests or symptoms (33). The frequency of potential interactions is higher than that of actual interactions, resulting in large discrepancies among study findings (24).

A valuable resource for detecting drug-drug interactions is a spontaneous reporting database (15,34). It currently uses several methods to detect possible drug-drug interactions (15,29,35,36). However, drug-drug interactions in general are rarely reported and information about the ADRs due to drug-drug interactions is usually lacking.

The aim of this study was to estimate the incidence of actual and potential drug-drug interactions in the national Spontaneous Reporting Database of ADRs in Croatia. Additionally, we assessed the clinical significance and seriousness of drug-drug interactions and their probable mechanism of action.

## Methods

### Data source

A retrospective observational study was performed from March 2005 to December 2008. Data were obtained from the Spontaneous Reporting Database of Pharmacovigilance Department of the Croatian Agency for Medicinal Products and Medical Devices (HALMED). Spontaneous Reporting Database uses VigiFlow, web-based Individual Case Safety Report management system that is specially designed for use by national centers in the World Health Organization Programme for International Drug Monitoring. Data elements for transmission of individual case safety reports are entered according to standardized ICH E2B format (International Conference on Harmonization of Technical Requirements for Registration of Pharmaceuticals for Human Use) (37).

ADR reporting is a legal obligation of every health care professional in Croatia, and patients are free to report as well (38,39). Reports are made in writing and sent by land mail, fax, or electronic mail using a prescribed form. Reporters are classified into the following groups: primary care physicians, secondary and tertiary care physicians, pharmacists, and patients. Drugs are grouped according to the Anatomical-Therapeutic-Chemical drug classification (ATC) and entered into the spontaneous reporting database. One report can describe one or more ADRs.

Data concerning the suspected ADRs were coded into the related Preferred Term and System Organ Class using the Medical Dictionary for Drug Regulatory Affairs (MedDRA) adverse drug reaction terminology (40).

### Data analysis

ADR reports were considered only if pharmacovigilance assessors evaluated the relatedness to the use of the suspected drug as possible, probable, or certain (41). We analyzed all reports involving at least two drugs suspected of causing an ADR. Drug-drug interactions were detected with online version of computerized interaction detection system Drug-Reax (42). To confirm Drug Reax results, we used online version of Stockley drug interaction program (43) and literature search of Medline.

For each report containing a potential drug-drug interaction, we verified if the description of the ADR corresponded to the interaction effect described in drug interaction programs and if it did it was considered actual (eg, first-dose hypotension of ACE inhibitors in patients receiving long-term treatment with ACE inhibitors and diuretics). Evaluation was made by a panel of five experts, including a pharmacist, clinical pharmacist, two physicians, and a clinical pharmacologist. They reviewed all the available information in the reports, and when necessary, the reporter was contacted for follow-up information. ADRs were considered serious if they resulted in one of the following outcomes according to the ICH E 2A (44) guidelines: death, life threatening condition, inpatient hospitalization, or prolongation of existing hospitalization, persistent or significant disability/incapacity, a congenital anomaly/birth defect, or other important medical event according to CIOMS V (Council for International Organizations of Medical Sciences, 2001) (45). Data were analyzed in respect to the total number of reports, demographic characteristics of the patients, classification of suspected drugs, System Organ Class, seriousness, and the source of reports. Medians (ranges) and percentages were used to present the data. ADR reports caused by actual drug-drug interactions were compared with ADR reports not caused by actual drug-drug interactions. The results were analyzed using the Mann-Whitney and χ^2^ test. Data analysis was done by the Predictive Analytics Software, version 18.0 (SPSS Inc., Chicago, IL, USA).

## Results

### Incidence of drug-drug interactions

From March 2005 to December 2008, HALMED received 2076 reports of ADRs, 1209 of which (58.2%) involved more than one drug. In 94 (7.8%) of these, ADRs were caused by actual drug-drug interactions, with 106 different pairs of concomitantly administered drugs. In 83 reports (88.3%), ADRs were caused by only one drug-drug interaction, in 10 reports (10.6%) by 2 drug-drug interactions, and in 1 report (1.1%) by 3 drug-drug interactions.

Potential drug-drug interactions were identified in 468 of 1209 reports (38.7%) involving more than one drug, and actual drug-drug interactions in 94 reports (20.8% of potential drug-drug interactions).

### Characteristics of actual drug-drug interactions

In 94 reports of actual drug-drug interactions, the age range of patients was 3 days-85 years (median 58 years). In 14 cases, patients were less than thirty years old, and all interacting drugs prescribed to them belonged to nervous system medications. On average, they were taking 4 medications, ranging from 2 to 12. Fifty-three patients were female and 41 were male.

Most ADRs belonged to the following System Organ Classes: gastrointestinal disorders (24.3%), nervous system disorders (14.5%), investigations (11.8%), and psychiatric disorders (11.2%) (Table 1). Most frequently reported MedRA Preferred Terms of drug-drug interactions were gastro-intestinal tract hemorrhage and muscular disorders.

**Table 1:** Distribution of adverse drug reactions (ADR) caused and not caused by actual drug-drug interactions (DDI) classified by Medical Dictionary for Drug Regulatory Affairs System Organ Class (MedDRA SOC) (40)

	No. (%) of ADRs	
MedDRA SOC	caused by actual DDIs*	not caused by DDIs^†^
Gastrointestinal disorders	37 (24.3)	625 (21.3)
Nervous system disorders	22 (14.5)	325 (11.1)
Investigations	18 (11.8)	96 (3.3)
Psychiatric disorders	17 (11.2)	123 (4.2)
General disorders and administration site conditions	16 (10.5)	324 (11.0)
Skin and subcutaneous tissue disorders	10 (6.6)	625 (21.3)
Blood and lymphatic system disorders	9 (5.9)	62 (2.1)
Muscle disorders	9 (5.9)	111 (3.8)
Vascular disorders	4 (2.6)	27 (0.9)
Respiratory, thoracic, and mediastinal disorders	2 (1.3)	187 (6.4)
Metabolism and nutrition disorders	2 (1.3)	37 (1.3)
Cardiac disorders	2 (1.3)	50 (1.7)
Reproductive system and breast disorders	1 (0.7)	43 (1.5)
Eye disorders	1 (0.7)	103 (3.5)
Renal and urinary disorders	1 (0.7)	42 (1.4)
Infections and infestations	1 (0.7)	22 (0.7)
Other	/	132 (4.5)

*94 reports contained 152 adverse drug reactions.

†1982 reports contained 2934 adverse drug reactions.

Fifty-three out of 94 ADR reports (56.4%) caused by actual drug-drug interactions were serious, 2 of which were fatal (3.8%), 12 were life threatening (22.6%), 23 led to hospitalization (43.4%), and 16 were serious according to the CIOMS V criteria (30.2%). Fatal cases were 69-year old woman who died from stomach and duodenal perforation due to additive gastrointestinal toxicity of amlodipine and indometacin and a 55-year old man who died from pancytopenia and sepsis developed from fluorouracil toxicity caused by concomitantly administered fluorouracil and leucovorin.

### Drugs most frequently involved in actual drug-drug interactions

The most frequent drugs involved in actual drug-drug interactions were cyclosporine (n = 15), warfarin (n = 9), and fluvastatin (n = 8) (Table 2). According to the ATC classification, the drugs involved in 34.0% of actual drug-drug interactions were nervous system medications (ATC group N). In most of these interactions, both interacting drugs belonged to nervous system medications (33 out of 39) and the most frequently interacting drug was paroxetine (Table 2). Interaction between lamotrigine and valproic acid caused serious rash with systematic involvement in one young man (23 years), two girls (4 and 14 years, respectively), and one boy (16 years) (Table 2).

**Table 2:** Drugs most frequently involved in actual drug-drug interactions (DDI)*

Drug (number of DDIs*)	Drug pairs (number of DDI combinations)	Most relevant reported ADRs	Mechanism of interaction
Cyclosporine (15)	cyclosporine - fluvastatin (6)	rhabdomyolysis, myopathy	inhibition of fluvastatin metabolism
	cyclosporine- amlodipine (3)	cyclosporine toxicity	inhibition of cyclosporine metabolism by amlodipine
	cyclosporine – methylprednisolone (2)	cyclosporine toxicity	decreased metabolism of cyclosporine
	cyclosporine – prednisone (1)	cyclosporine toxicity	decreased metabolism of cyclosporine
	cyclosporine- carvedilol (1)	cyclosporine toxicity	inhibition of cyclosporine metabolism
	cyclosporine- simvastatin (1)	rhabdomyolysis	inhibition of cytochrome P450-mediated metabolism of simvastatin and transporters
	cyclosporine- ciprofloxacin (1)	cyclosporine toxicity	decreased cyclosporine metabolism; pharmacodynamic antagonism
Warfarin (9)	warfarin-enoxaparin (2)	bleeding	additive anticoagulation
	warfarin- acetaminophen (1)	bleeding	inhibition of warfarin metabolism or interference with clotting factor formation
	warfarin -allopurinol (1)	bleeding	inhibition of warfarin metabolism
	warfarin-amoxicillin (1)	increased INR	unknown
	warfarin-atenolol (1)	increased INR	unknown
	warfarin-fluvastatin (1)	INR increased and bleeding	inhibition of warfarin metabolism
	warfarin- methylprednisolone (1)	anemia	unknown
	warfarin-simvastatin (1)	myalgia	competition for cytochrome P450 3A4-mediated metabolism
Fluvastatin (8)	fluvastatin -cyclosporine (6)	see above	
	fluvastatin -diclophenac (1)	gastrointestinal toxicity	inhibition of diclophenac metabolism
	fluvastatin -warfarin (1)	see above	
Paroxetine (8)	paroxetine - risperidone (4)	extrapyramidal ADRs, weight increase	concomitant use of paroxetine (potent CYP2D6 inhibitor) and risperidone (CYP2D6 substrate) has resulted in increased risperidone plasma concentrations and an increased risk of risperidone adverse effects.
	paroxetine - clozapine (1)	sedation, hypotension	decreased clozapine metabolism
	paroxetine- fluphenazine (1)	extrapyramidal ADRs	inhibition of cytochrome P450-mediated fluphenazine metabolism by paroxetine
	paroxetine-meloxicam (1)	hematochezia	At therapeutic doses SSRIs can block this reuptake of serotonin by platelets, leading to serotonin depletion, impairment of hemostatic function and increase the risk of bleeding
	paroxetine - tramadol (1)	palpitations, headache, dizziness	increased concentration of serotonin in the nervous system and periphery; inhibition of the CYP2D6-mediated formation of tramadol active metabolites (-)-M1 and (+)-M1 by paroxetine
Risperidone (7)	risperidone- paroxetine (4)	see above	see above
	risperidone- clozapine (1)	incontinence of urine	compete for metabolism by the cytochrome P450 isoenzyme CYP2D6 resulting in a reduction in the metabolism of the clozapine
	risperidone - haloperidol (1)	QT prolongation	additive effects on QT prolongation
	risperidone - quetiapine (1)	neuroleptic malignant syndrome	additive effects
sertraline (5)	sertraline - alprazolam (1)	neonatal respiratory distress syndrome	inhibition of cytochrome P450 3A-mediated alprazolam metabolism
	sertraline - amitriptyline (1)	sedation	inhibition of amitriptyline metabolism
	sertraline -lithium (1)	nausea and vomiting	additive effect
	sertraline - olanzapine (1)	weight increased	inhibition of olanzapine mechanism
	sertraline - zolpidem (1)	hallucinations	unknown
Valproic acid (5)	valproic acid- lamotrigine (4)	life-threatening rashes	decreased lamotrigine metabolism
	valproic acid - amitriptyline (1)	disorientation, amnesia, hallucinations	decreased amitriptyline plasma clearance
Acetylsalicylic acid (5)	acetylsalicylic acid -clopidogrel (3)	bleeding	inhibition of platelet aggregation
	acetylsalicylic acid -enoxaparin (1)	bleeding	decreased platelet function; decreased coagulation
	acetylsalicylic acid - ketoprofen (1)	gastrointestinal toxicity	additive gastrointestinal irritation

*Abbreviations: INR – International Normalized Ratio; SSRI – Selective Serotonin Reuptake Inhibitors; ADR – adverse drug reaction.

Twenty-seven percent of actual drug-drug interactions involved cardiovascular system medications (ATC group C). In most cases, the other interacting drug did not belong to the ATC group C (21 out of 35). We detected 16 drug-drug interactions involving hydroxymethylglutaryl-CoA reductase inhibitors (statins), 8 of which were caused by fluvastatin, and 4 drug-drug interactions involving simvastatin and atorvastatin each. The most frequent actual interaction was between fluvastatin and cyclosporine (n = 6) (Table 2).

There were 12.3% of actual drug-drug interactions that involved drugs belonging to antineoplastic and immunomodulating medications (ATC group L). Most of them involved cyclosporine (15 out of 21) (Table 2). Two cases of actual interactions involved methotrexate and non-steroidal anti-inflammatory drugs (ibuprofen, tenoxicam) and one involved methotrexate and omeprazole. The mechanism of these interactions involved decreased methotrexate clearance.

There were 10.9% of actual drug-drug interactions that involved medications affecting blood and blood forming organs (ATC group B), and more than half of the cases (9 out of 17) involved warfarin (Table 2). The most frequently reported actual drug-drug interaction in this group was between acetylsalicylic acid and clopidogrel (n = 3) (Table 2).

**Figure 1:**
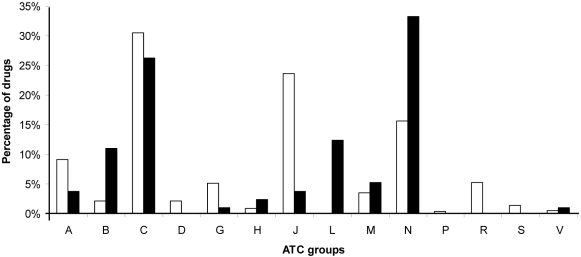
Distribution of suspected drugs according to Anatomical-Therapeutic-Chemical (ATC) drug classification in the group of adverse drug reaction reports not caused by drug-drug interactions (open bars) and the group of adverse drug reaction reports caused by drug-drug interactions (closed bars). ATC groups: A – Alimentary Tract and Metabolism; B – Blood and Blood Forming Organs; C – Cardiovascular System Drugs; D – Dermatologicals; G – Genito Urinary System and Sex Hormones; H – Systemic Hormonal Preparations, Excluding Sex Hormones and Insulins, Genito Urinary System and Sex Hormones; J – Antiinfectives for Systemic Use; L – Antineoplastic and Immunomodulating Medications; M – Musculo-Skeletal System; N – Nervous System; P – Antiparasitic Products, Insecticides and Repellents; R – Respiratory System; S – Sensory Organs; V – Various.

There were 15.8% of actual drug-drug interactions that involved drugs belonging to the musculo-skeletal system (ATC group M), alimentary tract, and metabolism (ATC group A), anti-infectives for systemic use (ATC group J), systemic hormonal preparations, excluding sex hormones and insulins, genito-urinary system and sex hormones (ATC group H), genito-urinary system and sex hormones (ATC group G), and various medicines group (ATC group V) (Figure 1).

**Figure 2:**
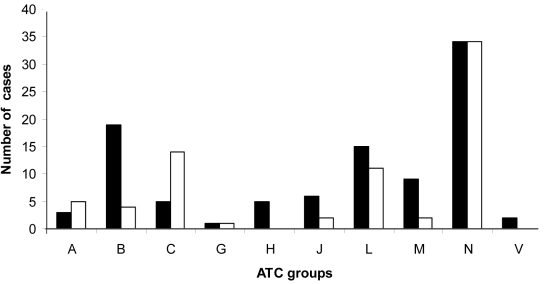
Distribution of serious (closed bars) and not serious (open bars) cases of actual drug-drug interactions in different Anatomical-Therapeutic-Chemical (ATC) drug classification groups. ATC groups: A – Alimentary Tract and Metabolism; B – Blood and Blood Forming Organs; C – Cardiovascular System Drugs; G – Genito Urinary System and Sex Hormones; H – Systemic Hormonal Preparations, Excluding Sex Hormones and Insulins, Genito Urinary System and Sex Hormones; J – Anti-infectives for Systemic Use; L – Antineoplastic and Immunomodulating Medications; M – Musculo-Skeletal System; N – Nervous System; V – Various.

Most actual drug interactions involved pharmacokinetic interactions (44.3%), 32.1% involved pharmacodynamic interactions, and 3.8% involved both pharmacokinetic and pharmacodynamic interactions. In 19.8% of actual drug-drug interactions, the mechanism was unknown. The proportion of serious cases caused by actual drug-drug interactions was highest in the following ATC groups: H and V, B, and J and M (Figure 2), and lowest in H ATC group (5 drugs) and V ATC group (2 drugs).

In 7 out of 94 ADR reports caused by actual drug-drug interactions, at least one of the interacting drugs was an over-the-counter drug. In 6 cases, it was acetylsalicylic acid and in 2 cases ranitidine. Moreover, 6 out of 7 ADRs involving over-the-counter drugs were serious. Two additional serious ADR cases involved drugs administered in the dose registered as over-the-counter (eg, ibuprofen 400 mg and omeprazole 10 mg).

### Reporters of adverse drug reactions involving actual drug-drug interactions

**Figure 3:**
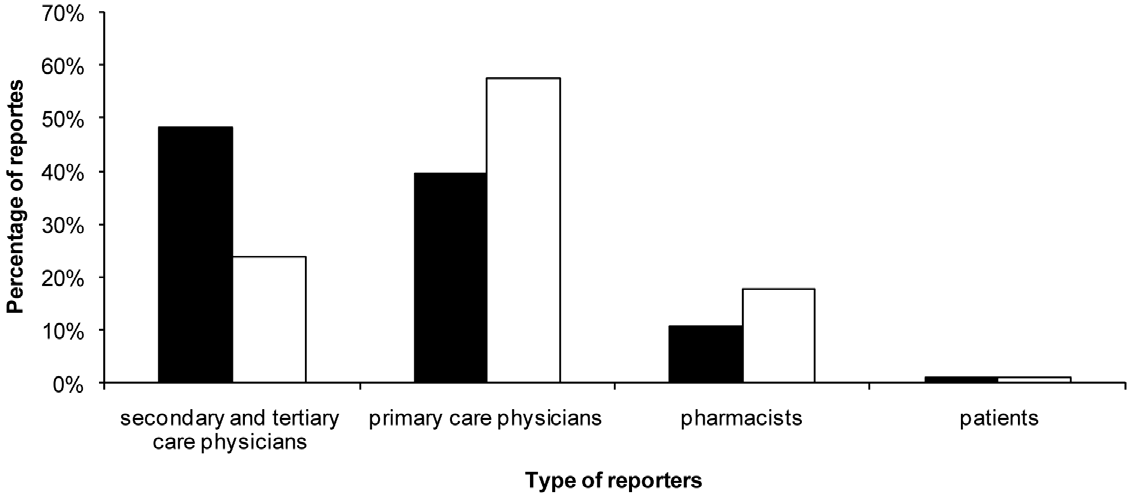
Distribution of reporters in the group of adverse drug reaction reports caused by drug-drug interactions (closed bars) and the group of adverse drug reaction reports not caused by drug-drug interactions (open bars).

ADRs caused by actual drug-drug interactions were in most cases reported by secondary and tertiary care physicians and ADRs not caused by actual drug-drug interactions by primary care physicians (Figure 3). The most serious ADR reports caused by actual drug-drug interaction were detected by secondary and tertiary care physicians (33 reports).

Actual drug-drug interactions were recognized by the reporter himself or herself in only 12 out of 94 reports. In the remaining reports, the reporter either did not state whether both interacting drugs were the suspected drugs or the box “ADR caused by drug-drug interaction” was not ticked. Reporters most frequently recognized actual drug-drug interactions in which one of the drugs was an anticoagulant/antiplatelet and least frequently drug-drug interactions in which one of the drugs was a psychiatric drug (only one case).

### Comparison of reports of adverse drug reactions caused and not caused by actual drug- drug interactions

The number of serious reports (53 out of 94) and the number of drugs (median 4, range 2-12) in therapy was significantly higher in the group of ADR reports caused by actual drug-drug interactions (*P* < 0.001) than among all other reports (580 out of 1982; median 2, range 1-14, respectively).

These groups did not differ significantly in sex distribution or patient age (Table 3), but did differ in the distribution of the most frequently reported ADRs according to MedDRA System Organ Class (Table 1). The greatest difference between ADR reports caused by actual drug-drug interactions and all other reports was observed in the following System Organ Classes: skin and subcutaneous tissue disorders (6.6% vs 21.3%), investigations (11.8% vs 3.3%), psychiatric disorders (11.2% vs 4.2%), blood and lymphatic system disorders (5.9% vs 2.1%), and respiratory, thoracic and mediastinal disorders (1.3% vs 6.4%) (Table 1).

**Table 3:** Comparison of adverse drug reaction (ADR) reports caused and not caused by actual drug-drug interactions (DDI)

Characteristic	Median (range) or number (%) of ADR reports*		*P*
	not caused by DDIs	caused by DDIs	
Number of drugs prescribed	2 (1-14)	4 (2-12)	<0.001
Patient age	55 y (1 days-93 years)	58 y (3 days-85 years)	0.115
Patients’ sex:			
female	1270 (64.1)	53 (56.4)	0.535
male	712 (35.9)	41 (43.6)	
Seriousness of ADR:*			
serious	580 (29.3)	53 (56.4)	<0.001
not serious	1402 (70.7)	41 (43.6)	

*Serious adverse drug reactions were those that resulted in the following outcomes according to the ICH E 2A (International Conference on Harmonization of Technical Requirements for Registration of Pharmaceuticals for Human Use) guideline (44): death, life threatening condition, inpatient hospitalization or prolongation of existing hospitalization, persistent or significant disability/incapacity, a congenital anomaly/birth defect, or other important medical event according to CIOMS V (Council for International Organizations of Medical Sciences 2001).

These two groups also differed according to the distribution of the most frequently suspected drugs according to ATC classification (Figure 1), and a significant difference was found for ATC groups J (*P* < 0.001), L (*P* < 0.001), B (*P* < 0.001), N (*P* = 0.009), R (*P* = 0.013), A (*P* = 0.025), and H (*P* = 0.029). The percentage of drugs belonging to N, L, B, and H ATC group was significantly higher in the group of actual drug-drug interactions reports (Figure 1).

## Discussion

Our study confirmed that the Spontaneous Reporting Database was a valuable resource for detecting ADRs caused by drug-drug interactions and the drugs leading to serious ADRs and deaths, thus emphasizing the areas that should be in the focus of health care education. Most of the studies investigating ADRs associated with drug-drug interactions were set in either hospital (3,10,29,46,47) or general practice setting (14,18,45-49). There was only a single study with the same methodology as ours. Leone et al (15) analyzed Italian spontaneous reporting database and concluded that it can be an important resource for detecting ADRs associated with the concomitant use of interacting drugs. Similar to us, they used all the cases that Drug Reax evaluated as possibly, probably, or certainly related to the use of suspected drugs (15).

The methodology of detecting drug-drug interactions may be a limitation of our study, since at the time of analysis not all drug-drug interactions were known and drug-drug interactions clinically unimportant for the majority of patients can be consequential for the individual with decreased enzyme activity. Further limitations include under-reporting of ADRs, and reports with missing information on concomitant drugs and comorbidities due to retrospective study design. According to some studies, fewer than 6% of all ADRs are reported (50). An additional limitation of our study could be the lack of denominator data such as the user population. Compared to other methodologies, we used more than one source of drug interaction-checking methods (43), which made our drug-drug interaction detection more sensitive, thus minimizing the possibility of unrecognized drug interactions. Also, consensus between experts was reached in all cases.

The percentage of reports involving actual drug-drug interactions was almost double in the Croatian than in Italian spontaneous reporting database (15). Probably because 60.9% of reports in the Italian database involved only one suspected drug without concomitant therapy, while in our study this figure was considerably lower (41.8%). The two studies showed no significant difference in the frequency of ADR reports caused by actual drug-drug interactions involving at least two drugs (*P* = 0.1446). However, our study had higher percentage of detected reports of actual drug-drug interactions, which could be explained by previously mentioned additional drug interaction-checking methods. Vonbach et al (51) placed Drug-Reax among the top four drug-drug interaction screening software programs, with 0.98 specificity and 0.71 sensitivity when compared with Stockley’s Drug Interactions, which is used as gold standard. Accordingly, our study had significantly higher percentage of reports involving at least one potential drug-drug interaction than the study by Leone et al (15). It had a comparable proportion of actual among potential drug-drug interactions (20.8%) with the study by Leone et al (21.7%) (15), as well as with studies conducted in hospital settings (14.0%) (52) and among geriatric outpatients (25.5%) (53). These findings strongly confirm that drug-drug interactions represent an important and frequent problem in clinical practice.

Variables such as age, polimedication, and the state of disease increase the probability of an interaction (16,22,33,54,55). The percentage of serious ADR reports and the number of drugs in our study was higher in reports on actual drug-drug interactions than among the remaining reports (15,29,56). Lin Chen-Fang et al (54) found that the prevalence of potential drug-drug interactions increased in a linear mode with increasing age and with the number of prescribed drugs (55). Furthermore, these two factors interacted to increase the risk (54). However, we did not find any significant age difference between the patients experiencing ADRs caused by actual drug-drug interactions and those experiencing ADRs not caused by actual drug-drug interactions. This could be explained by the difference in the drugs involved; more than a third of actual drug-drug interactions in our study were caused by nervous system medications (especially paroxetine, risperidon, valproic acid, and lamotrigine), which are more frequently used by younger patients than other ATC groups. Since nervous system medications were most frequently involved in actual drug-drug interactions and hardly ever recognized by the reporter, we identified a special need for alertness in this drug area.

The drug most frequently involved in actual drug-drug interactions in our study was cyclosporine, while in the study by Leone et al it was the combination of digoxin and diuretics (15). Cyclosporine has been particularly hazardous in transplant patients who often require lipid-lowering therapy (57,58). Co-administration of these two types of drugs requires great caution since it can result in serious ADRs including rhabdomyolysis, as has been previously reported by our research group (59).

Serious drug-drug interactions frequently involved antiplatelet (60), anticoagulant (56), and non-steroidal anti-inflammatory drugs (27,53,61,62) that caused gastrointestinal disorders. It is essential that health care providers recognize potentially interacting drug pairs in order to reduce the risk of drug-drug interactions and associated drug-related morbidity and mortality. In our study, reporters less frequently recognized actual drug-drug interactions, which could be a consequence of restricted access to available drug interaction databases or alerting drug-interaction systems and their limited availability. Reporters most frequently recognized drug interactions including anticoagulant and antiplatelet drugs and least frequently those including psychiatric drugs. This finding was also confirmed by Leone et al (15). Our results supported the findings of previous studies on the importance of prescribing-support systems and the need for additional education of health care providers in this area (63).

The main reporters of ADRs caused by actual drug-drug interactions were secondary and tertiary care physicians, while the main reporters of all ADRs were primary care physicians. This shows that most drug-drug interactions occur in hospital settings or that ADRs caused by actual drug-drug interactions are serious and more frequently require hospitalization.

The number of over-the-counter drugs involved in ADRs caused by actual drug-drug interactions was relatively high considering the fact that over-the-counter drugs should have a good safety profile. Therefore, pharmacists, as the main dispensers, should be specially alerted about potential clinically relevant interactions.

In conclusion, the Spontaneous Reporting Database was a valuable resource for detecting ADRs associated with drug-drug interactions. We obtained clinical evidence about ADRs associated with actual drug-drug interactions relevant for clinical practice, which involved nervous system medications (ATC group N), antiplatelet, anticoagulant, and non-steroidal anti-inflammatory drugs (in most serious cases of ADRs). Our findings confirm that ADR reports caused by actual drug-drug interactions are more serious and frequently require hospitalization. Therefore, it is essential to introduce prescribing-support systems and additional education of health care professionals.

## 
